# Editorial: Balanced and Unbalanced Immune Response to Dengue Virus in Disease Protection and Pathogenesis

**DOI:** 10.3389/fimmu.2022.835731

**Published:** 2022-02-11

**Authors:** Tineke Cantaert, Nolwenn Jouvenet, Sean A. Diehl

**Affiliations:** ^1^ Immunology Unit, Institut Pasteur du Cambodge, Pasteur Network, Phnom Penh, Cambodia; ^2^ Institut Pasteur, Université de Paris, CNRS UMR3569, Virus Sensing and Signaling Unit, Paris, France; ^3^ Department of Microbiology and Molecular Genetics, Translational Global Infectious Diseases Research Center, University of Vermont Larner College of Medicine, Burlington, VT, United States

**Keywords:** dengue, diagnostics, adaptive immunity, biomarkers, innate immunity

The ongoing COVID-19 pandemic has highlighted a central principle of the host immune response to RNA virus infections: while most infected patients remain asymptomatic or mild symptomatic, a portion of patients experience severe clinical symptoms, suggesting the existence of host immune factors that determine susceptibility to symptomatic disease. One well-known RNA virus infection that is characterized by differential susceptibility to symptomatic disease due to host immune determinants is dengue. A well-balanced, early immune response is protective for the host and leads to asymptomatic infection or self-limiting disease. Exacerbated and skewed immune responses, mostly observed during secondary heterotypic infection, can result in dengue hemorrhagic fever or dengue shock syndrome. The aim of this Research Topic was to group research papers that provide insights into the mechanisms leading to protection from disease or immunopathology upon dengue infection.

One of the largest obstacles to understand in depth the sequence of the immune response to dengue virus and the mechanisms behind an unbalanced immune response remain the absence of animal models representative for human disease (1). One step forward to address these issues is the revisitation by Rathore et al. of the old paradigm that dengue virus does not cause infection in immunocompetent mice. In this paper, the authors aimed to systematically test the ability of clinical isolates of DENV1-4 to induce replicating and systemic infection in wild type C57BL/6 mice and characterize the clinical outcome of infection. The authors found consistent and efficient viral replication in target organs and observed clinical symptoms that seemed to mimic human disease. Even though this proposed murine model for DENV can contribute to our understanding of immune-mediated pathology, limitations remain such as the reproducibility of utilizing clinical DENV isolates and the non-natural mode of infection *via* intraperitoneal injection. Future studies should be directed to understand the observed discrepancy between laboratory strains and clinical isolates, to assess the production of infectious virus in these models, and a reproducible method to induce either inapparent or more severe disease in order to identify correlates of protection.

To explore putative biomarkers of severe dengue disease and their potential sources in humans the next study in this issue focused on the type I interferon response (IFN-I), which is induced by a broad array of viruses. Plasmacytoid dendritic cells (pDCs) are rare immune cells that circulate in the blood. In response to viral infection, they secrete large amounts of IFN-I. These cytokines are responsible for the induction of a rapid and efficient antiviral state. They also stimulate B cell responses. Upasani et al. assessed IFN-I response and frequencies of circulating pDCs in a cohort of hospitalized Cambodian children undergoing acute DENV infection. They found that patients with higher viral loads had elevated plasma level of IFN-I and increased percentages of circulating pDCs. Analyzing IFN-I concentration in plasma with ultrasensitive digital ELISA and expression of IFN-I and IFN-related genes in PBMCs revealed that IFN-I response were reduced in critically-ill patients, as compared to patients with mild disease. Thus, a robust and rapid IFN-I response, likely generated by pDCs, seems to be beneficial for DENV patients. It would be of interest to investigate further the link between pDC-mediated IFN-I responses and anti-DENV antibody production, and the predictive value of IFN-I as biomarker of severe disease.

Indeed, from the public health perspective, the ability to identify dengue patients which will progress to severe dengue will improve patient management and intervention allocation in resource constrained countries. The research presented here by Pradeep et al. aimed to identify novel biomarkers of severe disease at hospital entry. The authors hypothesized that early secretion of cytokines by innate immune cells such as NKT cells, monocytes and granulocytes could be associated with an unbalanced immune response as observed in severe dengue cases. At admission, slightly lower, but significant percentages of circulating NKT cells, monocytes and granulocytes produced tumor necrosis factor (TNF)-α in dengue patients with warning signs compared to dengue patients without warning signs. A composite biomarker – IFN-γ+ NKT cells combined with interleukin (IL)-6+ granulocytes– could distinguish patients who progressed to worsened disease during hospitalization with a sensitivity of 90% and a specificity of 76%. However, the frequencies of these cells in blood are very low and hence interpretation should be with caution and how these parameters can translate into clinical practice remains to be determined. Further development of predictive biomarkers involves large scale multi-center translational studies to take these biomarkers from the bench to the clinic.

During secondary dengue infections, suboptimal levels of cross-reactive antibodies, mainly directed against the structural protein E, can drive antibody-dependent-enhancement of infection, resulting in more severe clinical signs than during primary infection (2). Moreover, high antigenic resemblance among co-circulating flaviviruses complicates serological analysis. To characterize better the diversity, specificity and temporal evolution of DENV-specific antibodies in infected patient sera, Falconi-Agapito et al. use a library of 9,072 linear peptides covering the entire proteome of dengue, as well as of the 2 related flaviviruses Zika and yellow fever viruses. Sera from symptomatic individuals from Peru, which is an endemic area, or from primary-infected travelers returning to Belgium, were analyzed across four or two time points, respectively. The strongest IgG responses were detected in the early convalescent phase and were targeting peptides located mainly in 4 viral proteins (C, prM, E and NS1). This IgG response was higher in secondary infections compared to primary ones. The magnitude of the IgM response was higher in primary infected travelers than in Peruvian patients, but the breadth and depth of the response was lower in this group compared with the endemic subjects. IgM and IgG exhibiting dengue-specific epitopes were identified. Validating the findings on larger cohorts of patients would be necessary. Upon validation, such peptide-based serological tests could be used to differentiate primary from secondary infection and would be easier to manufacture than conventional full-length recombinant protein assays.

Detection of prior dengue infection with high sensitivity and specificity remains challenging. Echegaray et al. evaluated the performance of two new lateral flow rapid diagnostic tests (RDTs) determining DENV seropositivity in samples from healthy volunteers non-endemic, endemic, and pediatric cohorts. The Dengvaxia dengue vaccine is approved for DENV-seropositive subjects 9 years of age and up whereas younger and dengue-naïve vaccinees are at increased risk of post-vaccination dengue disease; thus, rapid and accurate evaluation of serostatus would aid dengue vaccination efforts. One of the RDTs was based on standard DENV1-4 envelope (E) antigen, while the second contained as antigen recombinant DENV1-4 envelope (E) proteins with engineered glycosylation sites in the fusion loop to reduce false positives due to fusion loop binding as well as pre-adsorption with glycosylated ZIKV E to further reduce false positives in DENV/ZIKV-endemic areas. Performance of the first standard RDT was insufficient even with quantitative assessment, while the glycosylated E RDT exhibited acceptable sensitivity and specificity when evaluated quantitatively through pixel intensity analysis of the indicator strip on the lateral flow device. Performance of this new RDT was comparable to ELISA-based or gold standard neutralization tests currently available. This paper highlights that continual improvement of RDTs which are currently most often deployed to assess symptomatic patients could be field-adapted to be read on a device and eliminate the need and time required for laboratory analysis. Such advances will facilitate the use of RDTs to identify healthy, eligible candidates that can be safely vaccinated.

Besides antibodies, antigen-specific T cells have been shown by several groups to be induced by dengue infection and vaccination (3–8), but their role in protection against or risk for severe dengue remains controversial due to differences in factors including experimental protocols, study populations, or potential differential roles of subpopulations of these cells. Sanchez-Vargas et al. compared two T cell reactivation protocols in a pediatric dengue cohort using samples at timepoints before and after secondary dengue infection to ask if the dengue-specific T cell response pre-secondary infection would be changed upon secondary infection and if the response would correlate with clinical severity of secondary dengue. Brief *ex vivo* reactivation with peptides derived from structural or nonstructural DENV genes yielded low interferon (IFN)-γ responses and did not demarcate recent secondary infection or clinical severity. Instead culturing cells with non-structural peptides for up to two weeks yielded improved overall sensitivity with IFN-γ responses increased after infection in those subjects not requiring hospitalization. In this small study, NS-specific IFN-γ responses pre-secondary infection were higher in subjects who went on to require hospitalization after DENV2 secondary infection. The balance of serotype-specific or cross-reactive antibody responses and neutralization capacity thereof may be also important in clinical outcome (9). Though memory T cells are thought to be quickly reactivated these are a heterogenous mixture and ex vivo culture may better reveal the functional potential of convalescent post-primary DENV T cells.

Finally, simulation studies and mathematical models are being developed in an attempt to clarify the complex protective or disease-enhancing immune responses to primary or secondary dengue virus exposures (10). In this respect, Nguyen et al. adapted a model of affinity maturation by including interactions between B cells and Thelper cells and viremia dynamics. The authors simulated the immune responses after secondary heterotypic DENV infections to identify parameters that determine severity of disease. In their stochastic model, dengue-specific antibodies produced during the primary infection, together with lymphocyte counts, were important determinants of increased production of cross-reactive antibodies upon secondary heterotypic challenge associated with enhanced disease severity. Future applications include modeling of vaccination and vaccine efficacy scenarios.

This Research Topic highlights advances in *in vivo* pathogenesis models, more sensitive clinical immune activation assays, RDTs and biomarker studies which collectively can iteratively improve mathematical and clinical decision-making models towards prediction of risk or, more desirably, of vaccine protection against dengue disease, which threaten an estimated 100-400 million individual each year.

## Author Contributions

All authors contributed to the article and approved the submitted version.

## Funding

SD is supported by NIH grants U01AI141997, U01A1134582, P20GM125498, and R01AI107731. Flavivirus studies in the laboratory of NJ are funded by Institut Pasteur, CNRS and the Agence Nationale de la Recherche. TC is a HHMI/Welcome Trust international research scholar (208710/Z/17/Z) and is supported by NIH grant U01AI151758 – 01 and Institut Pasteur.

## Conflict of Interest

The authors declare that the research was conducted in the absence of any commercial or financial relationships that could be construed as a potential conflict of interest.

## Publisher’s Note

All claims expressed in this article are solely those of the authors and do not necessarily represent those of their affiliated organizations, or those of the publisher, the editors and the reviewers. Any product that may be evaluated in this article, or claim that may be made by its manufacturer, is not guaranteed or endorsed by the publisher.
